# Histone H3K27M Mutation Overrides Histological Grading in Pediatric Gliomas

**DOI:** 10.1038/s41598-020-65272-x

**Published:** 2020-05-20

**Authors:** Amal Mosaab, Moatasem El-Ayadi, Eman N. Khorshed, Nada Amer, Amal Refaat, Mohamed El-Beltagy, Zeinab Hassan, Sameh H. Soror, Mohamed Saad Zaghloul, Shahenda El-Naggar

**Affiliations:** 1 Children’s Cancer Hospital Egypt 57357, Tumor Biology Research Program, Research Department, Cairo, Egypt; 2 Children’s Cancer Hospital Egypt 57357, Department of Pediatric Oncology, Cairo, Egypt; 3 Children’s Cancer Hospital Egypt 57357, Department of Surgical Pathology, Cairo, Egypt; 4 Children’s Cancer Hospital Egypt 57357, Department of Radiology, Cairo, Egypt; 5 Children’s Cancer Hospital Egypt 57357, Department of Neurosurgery, Cairo, Egypt; 6 Faculty of Pharmacy, Helwan University, Department of Biochemistry and Molecular Biology, Cairo, Egypt; 7 Children’s Cancer Hospital Egypt 57357, Department of Radiotherapy, Cairo, Egypt; 8 National Cancer Institute, Cairo University, Department of Pediatric Oncology, Cairo, Egypt; 9 National Cancer Institute, Cairo University, Department of Surgical Pathology, Cairo, Egypt; 10 National Cancer Institute, Cairo University, Department of Radiology, Cairo, Egypt; 11 Faculty of Medicine, Cairo University, Department of Neurosurgery, Cairo, Egypt; 12 National Cancer Institute, Cairo University, Department of Radiotherapy, Cairo, Egypt

**Keywords:** CNS cancer, Cancer

## Abstract

Pediatric high-grade gliomas (HGG) are rare aggressive tumors that present a prognostic and therapeutic challenge. Diffuse midline glioma, H3K27M–mutant is a new entity introduced to HGG in the latest WHO classification. In this study we evaluated the presence of H3K27M mutation in 105 tumor samples histologically classified into low-grade gliomas (LGG) (n = 45), and HGG (n = 60). Samples were screened for the mutation in histone H3.3 and H3.1 variants to examine its prevalence, prognostic impact, and assess its potential clinical value in limited resource settings. H3K27M mutation was detected in 28 of 105 (26.7%) samples, and its distribution was significantly associated with midline locations (*p-value* < 0.0001) and HGG (*p-value* = 0.003). Overall and event- free survival (OS and EFS, respectively) of patients with mutant tumors did not differ significantly, neither according to histologic grade (OS *p-value* = 0.736, EFS *p-value* = 0.75) nor across anatomical sites (OS *p-value* = 0.068, EFS *p-value* = 0.153). Detection of H3K27M mutation in pediatric gliomas provides more precise risk stratification compared to traditional histopathological techniques. Hence, mutation detection should be pursued in all pediatric gliomas. Meanwhile, focusing on midline LGG can be an alternative in lower-middle-income countries to maximally optimize patients’ treatment options.

## Introduction

Central nervous system (CNS) tumors are the most common solid tumors in childhood^1^. Approximately 50% of CNS tumors are gliomas which display diverse histological behaviors and arise in various anatomical locations^2^. Histologically, gliomas have been classified into low- grade gliomas (LGG) (WHO grade I and II) and high-grade gliomas (HGG) (WHO grade III and IV)^3^. Unlike adults, LGG predominate in pediatrics with very good prognosis and extremely rare malignant progression. On the contrary, HGG are aggressive cancers and considered one of the most lethal tumors affecting the pediatric age group^4^. Anatomically, gliomas located in the cerebral hemispheres or in the infratentorial part of the brain are typically circumscribed. Hence, radical surgical resection is usually achievable and favorable outcomes are expected. Meanwhile, gliomas affecting midline structures such as brainstem and thalamus are generally difficult to resect due to their critical locations, and are thus associated with dismal prognosis^5,6^. In the last decade unprecedented efforts were made towards a better understanding of the molecular mechanisms underlying pediatric gliomas. High-throughput sequencing techniques have revealed new subgroups with distinct molecular, histological and clinical features^7,8^. Recent studies have reported a high frequency of two point mutations in the genes of the histone variants H3.3 *“H3F3A”*, and to a lesser extent H3.1 *“HIST1H3B”*, which results in substitution of lysine amino acid at position 27 with methionine (K27M) or glycine at position 34 with arginine or valine (G34V/R)^9,10^. Notably, these were the first reported histone mutations associated with human malignancies^11^. Further reports highlighted the association of K27M mutation with midline gliomas (MLG) and G34V/R mutation with gliomas of the cerebral hemispheres^7^. Mutation of the Lys 27 residue in the N-terminal tail of the H3 variants invokes disruption in post-translational modifications (methylation and acetylation) and could potentially alter the expression of oncogenes and tumor suppressor genes. Consequently, the histone H3K27M mutation has drawn substantial attention to examine its role in gliomagenesis^12,13^. In its latest version of CNS tumors classification, the World Health Organization (WHO) included a novel entity called “diffuse midline glioma, H3 K27M-mutant”. This entity is a lucid illustration of the new principle of “integrated diagnosis” that relies on the combination of the genotypic and phenotypic characteristics of the tumor. This new entity includes tumors that are characterized by a diffuse growth pattern, H3K27M mutation and a midline location. Radiologically confirmed diffuse intrinsic pontine gliomas (DIPGs) that harbor the mutation, which constitute 70 to 90% of DIPGs^9,14,15^, are now included within this category^16^. Despite numerous studies reporting on this specific mutation, there is scarce data on the incidence of H3K27M mutation and its impact on survival of pediatric patients in lower-middle-income countries (LMIC). In this study we screened for the K27M and G34V/R mutations in *H3F3A* and *HIST1H3B* variants in a cohort of histologically and anatomically diverse pediatric Egyptian glial brain tumor samples. We aimed to detect the prevalence of the mutation among each pathological and anatomical subgroup and evaluate its impact on the patients’ survival outcomes.

## Results

Clinical and histopathological characteristics of patients are shown in (Table 1). Immunohistochemical analysis classified tumor samples into glioma grade I, II, III and IV (Online resource 1). Among our patient cohort, 26% (28/105) were H3K27M mutant. Mutation was non-randomly distributed among anatomical and histological subgroups, and was significantly associated with midline location and high-grade histology (*p-value* = 0.001, *p-value* = 0.002 respectively). None of the 105 tumor samples harbored H3G34V/R mutation (Table 1). Among H3K27M mutant tumors, 82% (n = 23) were detected in the *H3F3A* gene and 17.8% (n = 5) in the *HIST1H3B* gene. *H3F3A* K27M mutant tumors were all MLG, and histologically classified as HGG (n = 18) and LGG (n = 5). On the other hand, *HIST1H3B* K27M mutant tumors were distributed as MLG (n = 4) and non-MLG (n = 1), and all of them were histologically classified as HGG. Mean age at diagnosis for patients with *H3F3A* mutant tumors was 9.4 years, compared to 5.9 years for patients with *HIST1H3B* mutant tumors.

**Table 1:** Summary of patients’ clinicopathological characteristics.

Patient Characteristics			Number (N = 105)	Percent
Gender		Male	59	56.19%
		Female	46	43.81%
Age at Diagnosis		Mean	7.54	
		<3	16	15.24%
		>= 3	89	84.76%
Tumor Site	MLG		(N = 74)	70.5%
		Thalamic	29	27.62%
		FBSG	25	23.81%
		DIPG	11	10.48%
		Suprasellar	4	3.81%
		Spinal	3	2.86%
		Basal Ganglia	1	0.95%
		Callosal	1	0.95%
	Non-MLG		(N = 31)	29.5%
		Cerebral Hemispheres	17	16.19%
		Cerebellar Hemispheres	14	13.33%
Pathology	HGG		(N = 60)	57.1%
		GBM,WHO GIV	34	32.38%
		Anaplastic astrocytoma, WHO GIII	18	17.14%
		Anaplastic ganglioglioma, WHO GIII	3	2.86%
		Anaplastic PXA, WHO GIII	2	1.90%
		HGA, NOS	2	1.90%
		Gliosarcoma, WHO GIV	1	0.95%
	LGG		(N = 45)	42.9%
		Pilomyxoid	10	9.52%
		Fibrillary astrocytoma, WHO GII	9	8.57%
		Ganglioglioma, WHO GII	7	6.67%
		LGA, NOS	7	6.67%
		Pilocytic astrocytoma, WHO GI	7	6.67%
		Ganglioglioma, WHO GI	3	2.86%
		DNET, WHO GI	1	0.95%
		Oligoastrocytoma, WHO GII	1	0.95%
Mutation	H3K27M		(N = 28)	26.7%
		H3F3A	23	21.9%
		HIST1H3B	5	4.76%
	H3G34V		(N = 0)	0%
		H3F3A	0	0
		HIST1H3B	0	0

### Frequency of H3K27M mutation according to anatomical location and pathologic grade

**Figure 1:**
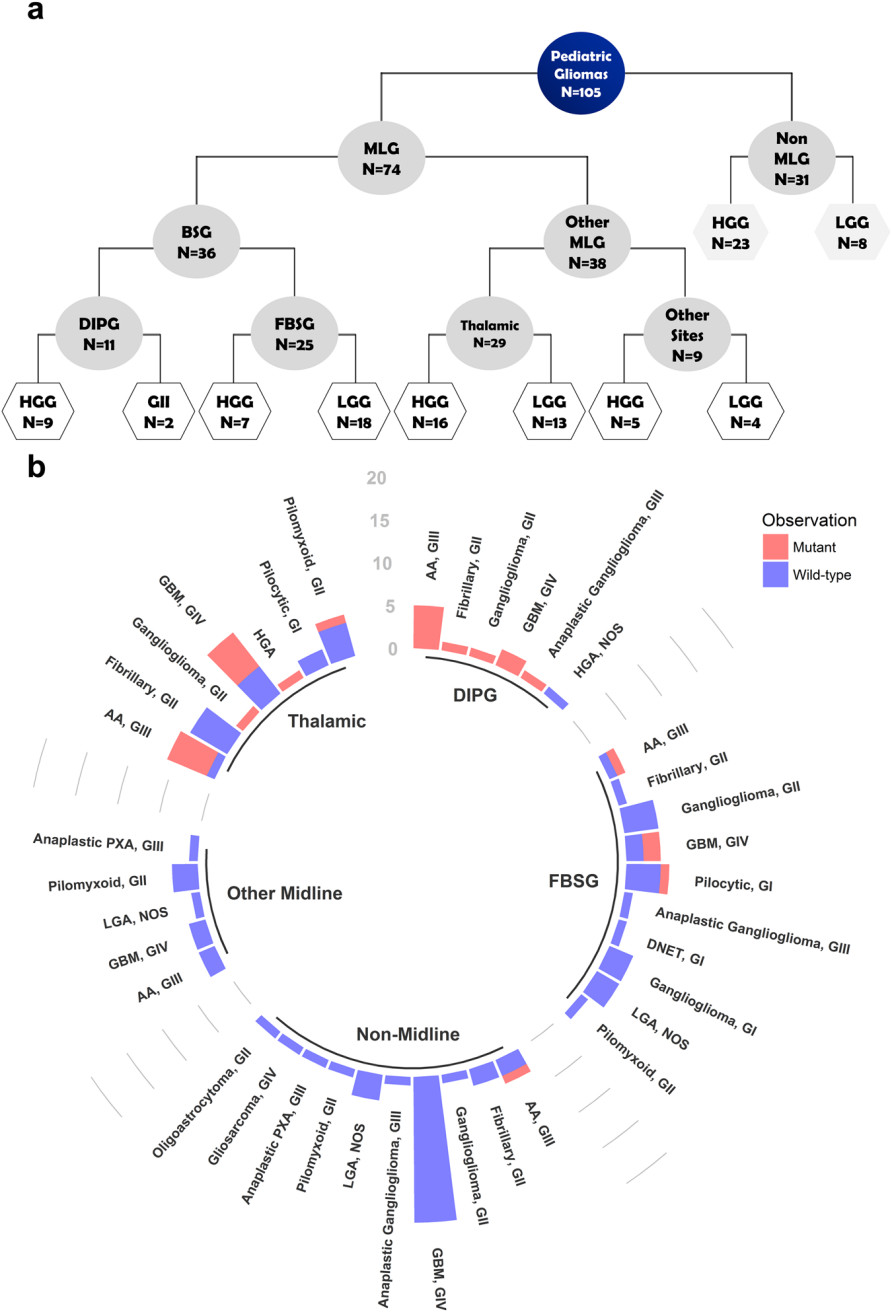
Cases and mutation distribution among the study cohort (**a**) Algorithm showing number of cases among different anatomical and histological subgroups (**b**) H3K27M mutation distribution among different anatomical and histological subgroups.

Among the midline-gliomas (MLG) (n = 74), H3K27M mutation was detected in 36.5% of cases (n = 27). Brainstem gliomas represented 48.6% of MLG (n = 36; DIPG = 11 and focal brain stem gliomas (FBSG) = 25) (Table 1). H3K27M mutation was detected in both DIPG (90.9%, n = 10) and FBSG (16%, n = 4) (Fig. 1b). Thalamic gliomas represented 39.2% (n = 29) of MLG, including 13 cases with H3K27M mutation. Only 3 cases had bilateral thalamic lesions, all of them were H3K27M mutant (Fig. 1b). On the other side, only one non-MLG (1/31) harbored the H3K27M mutation with a histopathological diagnosis of anaplastic astrocytoma, WHO GIII (Fig. 1b). This case was for a 12-year-old male patient with a lesion involving the right cerebellar peduncle and right cerebellar hemisphere. The patient was treated according to CCG-943 protocol which entails radiotherapy followed by 8 cycles of procarbazine, lomustine, and vincristine (PCV). He had a follow up period of 2.3 years since diagnosis with no death or progression.

Among histologically classified HGG (n = 60), 38.3% of cases had H3K27M mutation (n = 23). Glioblastoma, (GBM) WHO GIV, represented 56.7% (n = 34) of HGG. Although GBM were equally distributed between midline and non-midline locations, H3K27M mutation was only detected in midline tumors (n = 9/17) (Fig. 1b).

Anaplastic astrocytoma (AA), WHO GIII represented 30% (n = 18/60) of HGG. H3K27M mutation was detected in midline AA (n = 11/15) and non-midline AA (n = 1/3). Two remaining cases of mutant HGG were a case of anaplastic ganglioglioma and another case of HGG, not otherwise specified (NOS) (Fig. 1b). In histologically classified LGG (n = 45), H3K27M mutation was detected in 11.1% (n = 5) cases. One case was a pilocytic astrocytoma WHO GI, and 4 cases were WHO GII (Fig. 1b).

### Impact of H3K27M mutation on clinical outcome

Univariate Cox proportional hazards analysis for age, tumor site, histological grade and H3K27M mutation identified histological grade and mutation status to have a significant impact on both overall survival (OS) and event-free survival (EFS) *(p-value* < *0.0001)* (Table 2). Multivariate Cox regression further confirmed the prognostic value of H3K27M mutation; OS *(p-value* = *0.003)*, and EFS *(p-value* = *0.002)*, along with the pathological grade; OS *(p-value* < *0.0001)*, and EFS *(p-value* = *0.002)*, (Table 2). The median OS for *H3F3A* mutant cases was 9.4 months compared to 12.6 months for *HIST1H3B* mutant cases (*p-value* = 0.435).

**Table 2:** Univariate and multivariate Cox regression analysis.

Variables	OS				EFS			
	No. of events/No. of cases	HR	95% CI	*p-value*	No. of events/No. of cases	HR	95% CI	*p-value*
**Univariate Cox regression analysis**								
Age	105	1.001	0.94–1.06	0.963	105	0.98	0.92–1.03	0.51
Tumor Site	105				105			
Non-Midline	15/31	1	—	—	23/31	1	—	—
Midline	40/74	1.626	0.894–2.955	0.100	48/74	1.068	0.648–1.758	0.797
Histology	105				105			
LGG	12/45	1	—	—	21/45	1	—	—
HGG	43/60	4.86	2.48–9.55	<0.0001*	50/60	2.73	1.63–4.57	<0.0001*
H3K27M Mutant	105				105			
Wild-Type	32/77	1	—	—	45/77	1	—	—
Mutant	23/28	3.44	1.96–6.04	<0.0001*	26/28	2.78	1.69–4.58	<0.0001*
**Multivariate Cox regression analysis**								
Histology	105				105			
LGG	12/45	1	—	—	21/45	1	—	—
HGG	43/60	4.006	1.99–8.03	<0.0001*	50/60	2.32	1.36–3.95	0.002
H3K27M Mutant	105				105			
Wild-Type	32/77	1	—	—	45/77	1	—	—
Mutant	23/28	2.41	1.35–4.3	0.003	26/28	2.23	1.33–3.74	0.002

Estimated hazard ratio for overall and event-free survival with 95% confidence interval and *p-value* of the likelihood ratio test.

Abbreviations: OS = overall survival. EFS = Event- free survival. HR = hazard ratio, CI = confidence interval.

**Figure 2:**
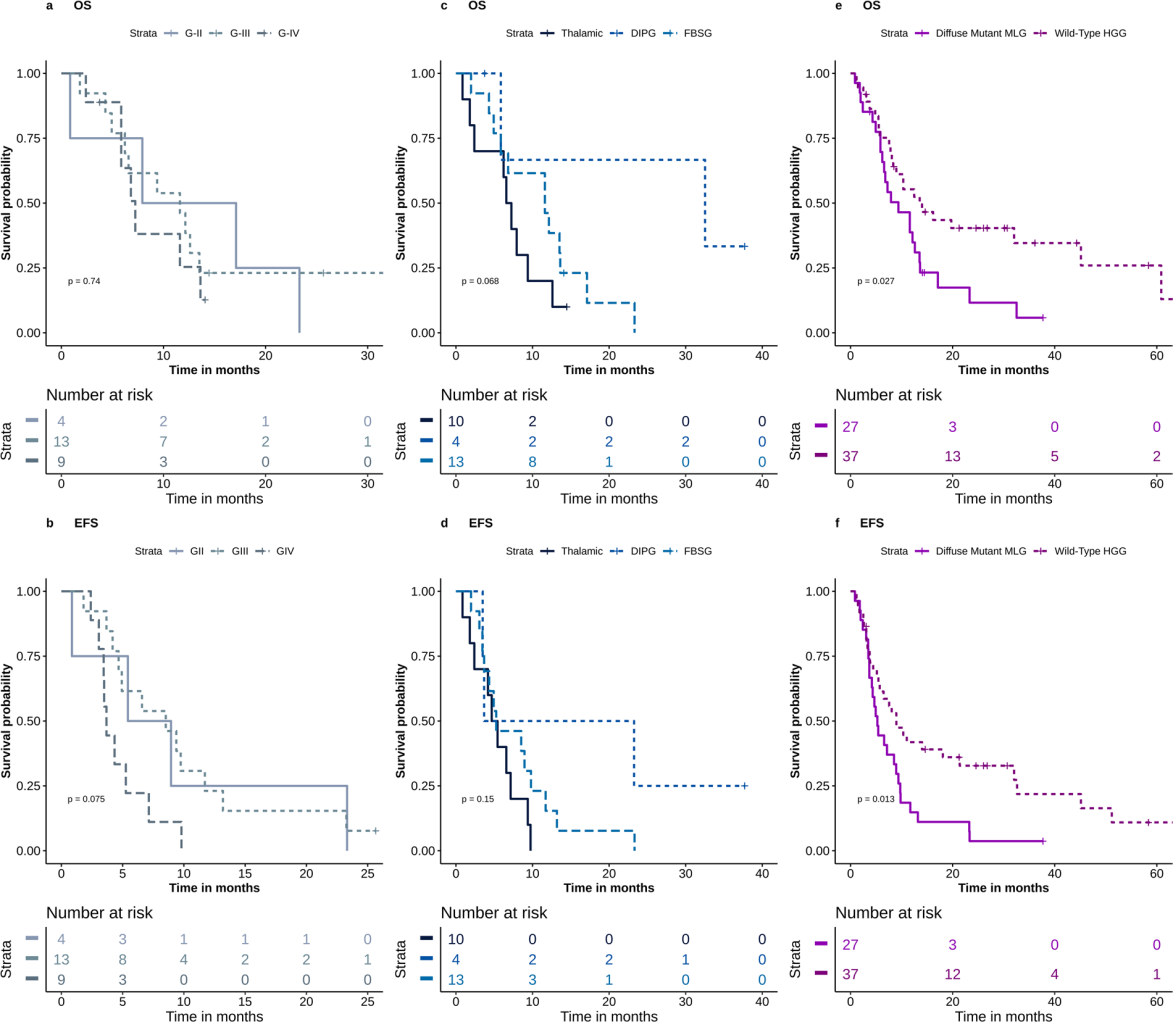
OS and EFS for different subgroups. (**a**) OS for mutant GII vs. GIII vs. GIV patients (**b**) EFS for mutant GII vs. GIII vs. GIV patients (**c**) OS for mutant FBSG vs. DIPG vs. thalamic gliomas (**d**) EFS for mutant FBSG vs. DIPG vs. thalamic gliomas (**e**) OS for diffuse mutant MLG vs. wild-type HGG (**f**) EFS for diffuse mutant MLG vs. wild-type HGG.

Next the impact of H3K27M mutation on survival among different histological subgroups was assessed. The mutation had a significant impact on the OS *(p-value* = 0.001) in LGG (Online resource 2a) while in HGG, the presence of H3K27M mutation was significantly correlated with inferior outcome in both OS (*p-value* = 0.035) and EFS (*p-value* = 0.012) (Online resource 2c and d respectively). The OS and EFS for mutant tumors did not differ significantly according to histologic grade (median OS of 7.9, 11.6 and 7.2 months for GII, GIII and GIV, respectively, *p-value* = 0.736 and median EFS of 5.4, 8.5 and 3.6 months for GII, GIII and GIV, respectively, *p-value* = 0.075) (Fig. 2a and b respectively). Meanwhile, histologic grades significantly correlated with survival outcome in wild-type tumors (median OS of 89.5 months, 12.4 months and 13.5 months for GII, GIII and GIV, respectively, *p-value* = 0.023) (Online resource 2b). The mutation retained its impact on survival across different anatomical sites, with no significant difference between mutant DIPG, FBSG, and thalamic tumors (the median OS for DIPG = 6.5, FBSG = 32.5 and thalamic = 11.6 months, *p-value* = 0.068, median EFS of 4.6, 3.6 and 5.3 months, respectively, *p-value* = 0.153), (Fig. 2c and d respectively). Patients with mutant MLG had inferior survival outcomes when compared to wild-type HGG; OS (*p-value* = 0.027) (Fig. 2e), EFS (*p-value* = 0.013), (Fig. 2f). The poor impact of H3K27M mutation on survival was evident in thalamic gliomas compared to wild type; OS (*p-value* = 0.006), (Online resource 2e) and EFS (*p-value* = 0.003), (Online resource 2 f), while it was not tested in DIPGs due to low number of wild-type cases.

## Discussion

Somatic mutations in histone H3.3 and H3.1 variants (*H3F3A* and *HIST1H3B*) were first identified in pediatric HGG and afterwards in chondrosarcomas and giant cell tumors of bone in adolescents. This missense mutation causes an amino acid substitution at a crucial location on the histone H3 N-terminal tail, affecting epigenetic regulation of gene expression^17,18^. Accordingly, we evaluated 105 pediatric gliomas for point mutations in the most common H3 variants (*H3F3A* and *HIST3H3B*). Tumor samples were selected to include all available brainstem gliomas along with other MLG and non-MLG.

H3K27M mutation was detected in 26% of our cohort. The mutation was significantly associated with high-grade histology and midline tumors. The presence of mutation was correlated with inferior outcome compared to wild-type tumors, regardless of their histologic grading or anatomical location. H3.1 K27M mutant tumors are mostly restricted to the pons, usually seen in very young children, frequently accompanied by other mutations such as ACVR1^19,20^ and associated with slightly longer EFS^14^. On the other hand, H3.3 K27M mutant tumors are seen in almost two-thirds of DIPG cases, as well as other midline HGG. They are more common in school-age children, and have dismal outcome, independent of location^8^. In contrast, G34V/R mutant tumors commonly arise in adolescents and young adults^9,10,21^. The association of different mutations with age potentially explains the absence of such mutation in our cohort given the age distribution of our cases. G34V/R mutant tumors are typically restricted to cerebral hemispheres and are not seen in midline locations^7,22,23^. This location specificity of histone-mutant tumors suggests that HGG harboring H3K27M and G34V/R mutations may arise from different cells of origin, or the same cell at different stages of differentiation, and are principally distinct diseases^23^. Moreover, previous studies have highlighted the association of histone mutations with anatomical tumor location, age of patients, and overall prognosis^4,8,24^. For instance, H3K27M mutant tumors are known to arise predominantly in midline structures such as the thalamus, pons, and spinal cord.

While our GBM cases were equally distributed between midline and non-midline locations, H3K27M mutations were only detected in midline GBM and were coupled with a dismal prognosis. Consistent with our findings, Korshunov *et al*. found that pediatric H3K27M mutant tumors in midline locations, including the brainstem and thalamus were associated with very poor survival^25^.

Our study illustrates the similarities in *H3F3A* and *HIST1H3B* genes mutations in MLG among populations with different socioeconomic environment and genetic background. Chen *et al*. raised the possibility of different epigenetic histone methyltransferase mutations according to race and ethnicity^26^. A few publications have illustrated that epigenetic modifications can be changed through diet, exercise, and other environmental factors. For example, Al-Kzayer *et al*. were able to demonstrate different frequencies of genetic and epigenetic mutation in leukemogenesis in Arab Asian children when compared to Western, Taiwanese and Japanese children^27,28^.

In our cohort, the negative prognostic impact of H3K27M mutation was maintained across all histological grades, whether low or high-grade. Moreover, there was no predictive power for the anatomical location (thalamic vs. DIPG vs. FBSG) on the survival outcome of mutant MLG, consistent with previous reports indicating the presence of H3K27M mutation as an independent negative prognostic marker in DIPG^9,15^ and thalamic gliomas^29,30^. Of interest, studies on adult midline HGG showed similar association between H3K27M mutation and poor survival in the brainstem, but not in thalamic tumors^31,32^.

In agreement with results from other studies^33,34^ our LGG cases had better survival rates with 73.3% of patients alive at end of the study with a median follow up of 25.7 months. However, all LGG patients who were H3K27M mutant had significantly lower survival with a median OS of 17.1 months, compared to the median OS exceeding 3 years (median OS not reached) in the wild-type group. Thus, it is reasonable to suggest that in histologically classified LGG, H3K27M mutant tumors should be treated more aggressively.

Different histological subtypes were included in our cohort with variable proportions. In general, around 50% of brain tumors are gliomas and mixed glioneuronal tumors with predominant low-grade histology in the pediatric age group^35^. In a previous report from our center, 893 cases of pathologically confirmed CNS tumors were diagnosed between July 2007 and December 2013 with gliomas and glioneuronal tumors comprising 42.7% (n = 381) of the total cohort^36^. During our study period, total number of same tumor types are almost doubled (unpublished data). Hence, the sample size of our cohort is not representative for the whole patient population treated in our center in the same period. Accordingly, the reported frequencies of different pathological subtypes do not reflect the real incidence of such tumor subtypes.

The studied cohort showed the significant impact of H3K27M mutation on both OS and EFS, thereby highlighting the potential value of routine screening for the mutation as a part of the glioma diagnostic panel. These histone mutations provide clinicians with better insights into patients’ prognosis, and help prioritizing and tailoring the management of pediatric malignant gliomas. Sequencing technologies are well established and fairly accessible in high-income countries, however their cost and labor-intensity are major challenges to their expanded use in LMIC. This has a direct impact on the quality of care for pediatric glioma patients in such regions since access to accurate diagnosis is a pillar of long-term survival and improved quality of life^37^. Hence, H3K27M mutation detection has become of particular importance in resource-limited centers where the use of the limited treatment resources must be optimized. This will have the most significant impact on clinical decisions in cases histologically classified LGG cases and arising from a midline structure. In such cases changing treatment plan will be a necessity because conventional chemotherapy modalities will be considered as a consumption of both time and resources. Moreover, early detection of mutation will give proper insight into the expected

disease progression, thereby allowing better palliative care and avoid unwarranted costly scans for progressed patients. This study is the first report on histone mutations in pediatric gliomas in Egypt – a LMIC – that coincides with those reported from international studies^38^. Exploring the prevalence and impact of histone mutations in other LMIC populations and other races is warranted.

## Conclusion

Our study confirms the prognostic significance of H3K27M mutation in pediatric gliomas and its strong association with high-grade histology and midline locations. Although the trend of poor prognosis was preserved in HGG compared to LGG, the presence of H3K27M mutation identified cases with worse outcome within each of these groups. This highlights the fact that H3K27M status is a very important supplement to histological grading of gliomas for appropriate clinico-pathological diagnosis. Testing for H3K27M mutations should be pursued in all pediatric midline gliomas independent of their histologic appearance or grade. Despite an apparently higher cost and longer diagnostic turnaround time needed, adopting the principle of “integrated” diagnosis for pediatric gliomas using both genotypic and phenotypic characteristics of the tumor would eventually be more beneficial and cost-efficient in resource-limited settings.

## Materials and Methods

### Patient cohort and tissue samples

We screened 105 tumor samples of pediatric cases diagnosed with glial and glioneuronal tumors at Children’s Cancer Hospital Egypt − 57357 (CCHE) during the period from January 2008 to June 2018, with an average follow up period of two years. The cohort included all available biopsied brain stem lesions; FBSG (n = 25) and DIPG (n = 11), in addition to other MLG (n = 38) and non-MLG (n = 31), (Fig. 1a). Samples were collected after the approval of the CCHE Institutional Review Board (IRB) for Human Research.

### Histopathological diagnosis

All formalin-fixed paraffin-embedded (FFPE) tissue blocks were obtained from the pathology department at CCHE. Cases were reviewed by a neuropathologist to confirm the pathological diagnosis and histological grading according to the WHO guidelines.

Tumors with WHO grade I or II were considered as low-grade tumors while grade III or IV were considered as high-grade tumors. Gangliogliomas displaying atypical histomorphologic features of increased cellularity, increased mitotic activity, Ki-67 proliferation index and p53 staining percentage, were considered as grade II after ruling out the presence of anaplastic features^39–43^.

### DNA isolation

DNA was isolated from 20 µm sections from each FFPE tumor block. DNA extraction was performed by QIAamp DNA FFPE tissue kit (Qiagen) according to the manufacturer’s recommendations except for deparaffinization that was performed via 2 rounds of xylene incubation, followed by rehydration with absolute ethanol. DNA concentration was measured by Infinite 200 PRO NanoQuant (Tecan) according to the manufacturer’s instructions. A total of 50–100 ng of DNA were used in the subsequent experiments.

### Targeted sequencing of H3F3A and HIST1H3B

Template DNA was amplified using *H3F3A* primers which were designed to cover the region encoding Lys27 and Gly34 in Histone H3.3 (forward: TGGCTCGTACAAAGCAGACT, reverse: ATGGATACATACAAGAGAGACT). *HIST1H3B* primers amplified the region encoding Lys27 and Gly34 in Histone H3.1 (forward: GTTTTGCCATGGCTCGTACT, reverse: AAGCGAAGATCGGTCTTGAA). PCR products were separated on 2% agarose gel then purified using Gene JET PCR Purification kit (ThermoFisher Scientific). Sanger sequencing was performed on the amplified DNA for detection of H3 (K27M) and (G34V/R) mutations, using the 3500 Series Genetic Analyzers (Applied Biosystems). Sequencing data was generated using Gene Mapper Software v5.0 (Applied Biosystems). Sequencing chromatograms were edited and visualized with Snap Gene software (from GSL Biotech; available at snapgene.com).

### Statistical analysis

Descriptive analysis for patients’ characteristics was reported in counts and percentages for categorical data, and means for continuous data. Pearson’s Chi square test was used to compare mutation distribution among different pathological and anatomical sites. Survival estimates were reported as OS and EFS. Time to event was calculated as duration from the time of diagnosis till time of recurrence, progression or death. Survival analysis was tested using Cox regression analysis and Kaplan–Meier method, log-rank test was used to compare between survival plots where *p-value* < 0.05 was considered significant. Statistical calculations were carried out using R v3.3.2, using survival^44^, ggplot2^45^, tidyverse^46^, viridis^47^ and xlxs^48^.

### Institutional review board statement

The study was performed in accordance with the declaration of Helsinki and experimental protocols were revised and approved by the IRB at CCHE on the 15^th^ of April, 2019.

### Informed consent statement

The IRB at CCHE has approved the waiver of patient consent form because the study used archived pathological samples and analysis performed on the samples does not affect patient well-being in any way. Patient confidentiality is maintained at all time in accordance with CCHE policies.

## Supplementary information

Supplementary Information.

## Data Availability

Data sharing not applicable to this article as no datasets were generated or analyzed during the current study.
