# Supplementary material for: Histone H3K27M Mutation Overrides Histological Grading in Pediatric Gliomas
Source: Sci Rep. 2020 May 20;10:8368. doi: 10.1038/s41598-020-65272-x (PMC7239884; doi:10.1038/s41598-020-65272-x)
Supplement: Supplementary file 1 — Supplementary Information. [file 41598_2020_65272_MOESM1_ESM.pdf]

Histone H3K27M Mutation Overrides Histological Grading in Pediatric Gliomas

Authors

Amal Mosaab[1], Moatasem El-Ayadi[2,8], Eman Khorshid[3,9], Nada Amer[1], Amal Refaat[4,10], Mohamed El-Beltagy[5,11], Zeinab Hassan[6], Sameh H. Soror[6], Mohamed Saad Zaghloul[7,12] and Shahenda El-Naggar[1*]

Affiliations

[1]Children's Cancer Hospital Egypt 57357, Tumor Biology Research Program, Research Department, Cairo Egypt

[2]Children's Cancer Hospital Egypt 57357, Department of Pediatric Oncology, Cairo Egypt

[3]Children's Cancer Hospital Egypt 57357, Department of Pathology, Cairo Egypt

[4]Children's Cancer Hospital Egypt 57357, Department of Radiology, Cairo Egypt

[5]Children's Cancer Hospital Egypt 57357, Department of Neurosurgery, Cairo Egypt

[6]Faculty of Pharmacy, Helwan University, Department of Biochemistry and Molecular Biology, Cairo, Egypt

[7] Children's Cancer Hospital Egypt 57357, Department of Radiotherapy, Cairo Egypt

[8]National Cancer Institute, Cairo University, Department of Pediatric Oncology, Cairo, Egypt

[9]National Cancer Institute, Cairo University, Department of Pathology, Cairo, Egypt

[10]National Cancer Institute, Cairo University, Department of Radiology, Cairo, Egypt

[11]Faculty of Medicine, Cairo University, Department of Neurosurgery, Cairo, Egypt

[12]National Cancer Institute, Cairo University, Department of Radiotherapy, Cairo, Egypt

Correspondence*:

Dr. Shahenda Mahmoud El-Naggar,

E-mail address: shahenda.elnaggar@57357.org

ORCID ID: 0000-0001-6465-9881

Research Department, Children's Cancer Hospital Egypt 57357, 1 Seket Al-Emam Street, Cairo, P.O box 11441, Egypt.

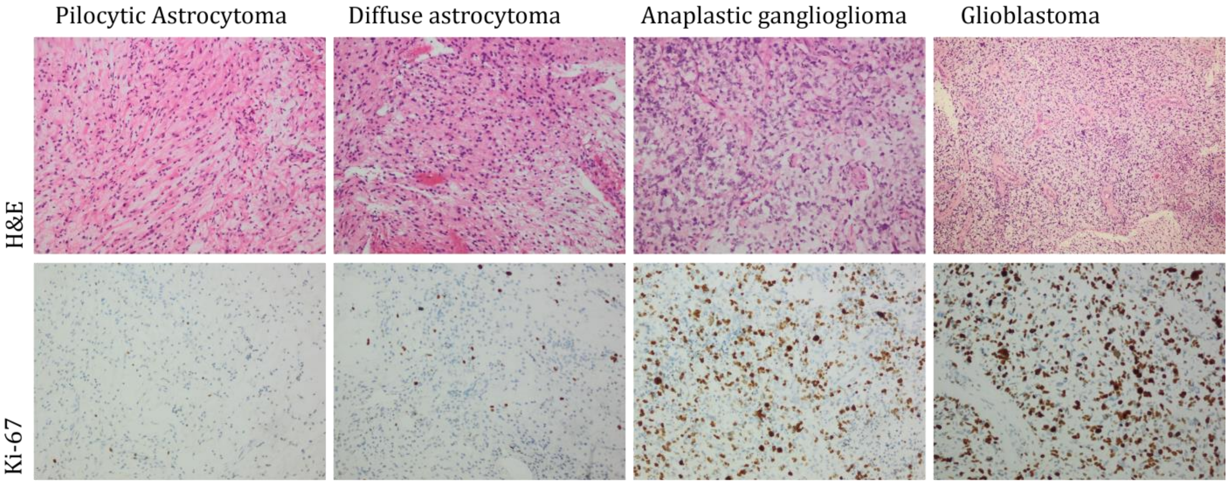

Supplementary figure 1 Immunohistochemistry for different types of astrocytic tumors with their corresponding Ki-67 proliferation indices. Glioblastoma, WHO grade IV, showing high Ki-67 labelling index about 70%. Anaplastic ganglioglioma, WHO grade III, with high Ki-67 labelling index about 30%. Diffuse astrocytoma, WHO grade II and Pilocytic astrocytoma, WHO grade I, with low Ki-67 labeling index about 5%.

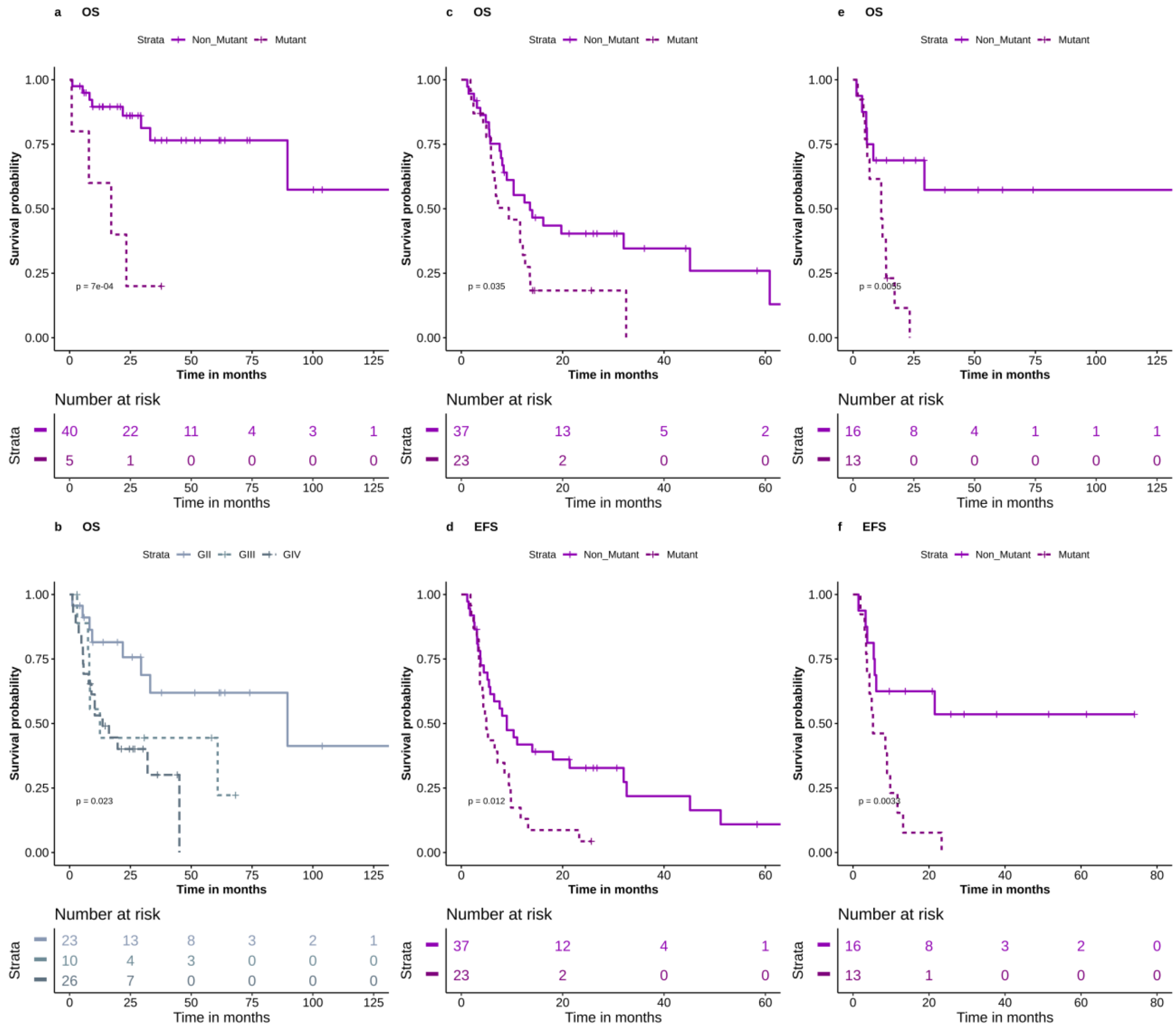

Supplementary Figure 2: OS and EFS for different subgroups.
(a) OS for LGG, mutant vs. wild-type
(b) OS for wild-type GII vs. GIII vs. GIV patients
(c) OS for HGG, mutant vs. wild-type
(d) EFS for HGG, mutant vs. wild-type
(e) OS for thalamic gliomas, mutant vs. wild-type
(f) EFS for thalamic gliomas, mutant vs. wild-type
